# Mechanical Properties and Economic Analysis of Fused Filament Fabrication Continuous Carbon Fiber Reinforced Composites

**DOI:** 10.3390/polym16182656

**Published:** 2024-09-20

**Authors:** Damira Dairabayeva, Ulanbek Auyeskhan, Didier Talamona

**Affiliations:** 1 Institute of Smart Systems and Artificial Intelligence (ISSAI), Nazarbayev University, Astana 020000, Kazakhstan; damira.dairabayeva@nu.edu.kz (D.D.); u.auyeskhan@astanait.edu.kz (U.A.); 2 Department of Intelligent Systems & Cybersecurity, Astana IT University, Astana 020000, Kazakhstan; 3 Department of Mechanical and Aerospace Engineering, Nazarbayev University, Astana 020000, Kazakhstan

**Keywords:** polymer-matrix composites, continuous carbon fibers, fused filament fabrication, tensile properties, flexural tests, cost/time analysis, additive manufacturing

## Abstract

Additive manufacturing of composites offers advantages over metals since composites are lightweight, fatigue and corrosion-resistant, and show high strength and stiffness. This work investigates the tensile and flexural performance of continuous carbon-fiber reinforced (CCF) composites with different guide angles and number of layers. The cost and printing time analyses were also conducted. Tensile specimens with a contour-only specimen and one CCF layer with a 0° guide angle exhibited nearly comparable strength values. Increasing the number of CCF layers enhances the tensile properties. For the identical cost and reinforcement amount, 0°/0° provides a higher tensile strength and elastic modulus compared with 15°/−15°. The same phenomenon was observed for 15°/0°/−15° and 0°/0°/0°. The samples with one and two reinforcement layers had similar stiffness and maximum load values for flexural tests. For the samples with four layers, there was a considerable improvement in stiffness but a minor decrease in the maximum load.

## 1. Introduction

Additive manufacturing (AM) of carbon fiber-reinforced polymer composites (CFRP) provides significant advantages over metals since composites are lightweight, possess corrosion and fatigue resistance, and demonstrate high strength and stiffness [1]. CFRPs currently receive considerable attention in the aerospace and electronics industries. The AM processes used for manufacturing CFRPs include fused filament fabrication (FFF), stereolithography, selective laser sintering, and laminated object manufacture [2]. Among the AM technologies, FFF, an extrusion-based process that uses thermoplastic filaments as material, is one of the most widely used processes for 3D printing polymers [3]. AM CFRPs are usually divided into two main groups based on the reinforcement type, which are continuous carbon fiber-reinforced polymers and short carbon fiber-reinforced polymers.

On the one hand, the most commonly utilized reinforcement consists of short or discontinuous fibers, primarily because they are easier to implement. Typically, only an extruder suitable for discontinuous carbon fibers is required. These reinforcements also enhance the mechanical performance of thermoplastics. However, the length of fibers can restrict the advantages of the short fiber reinforcement. The discontinuous CFRPs are produced by embedding milled or chopped fibers with a length of a few hundred microns into the thermoplastic filament [3]. Fibers shorter than the diameter of the nozzle by an order of magnitude should be used to prevent nozzle clogging. This issue is relatively common with this technology [3,4]. On the other hand, continuous carbon fibers are prepregs that are embedded by simultaneous injection of the polymer and fiber filaments into the nozzle; this technology is also known as coaxial extrusion or co-extrusion. The composite created is deposited on the component to produce composite parts [4,5].

Few studies compared the tensile performance of short and continuous carbon fiber-reinforced composites. Dickson et al. [6] compared three types of continuous fiber reinforcement in nylon composites and observed carbon fiber exhibiting the highest tensile and flexural strength, followed by glass and, lastly, Kevlar^®^, DuPont de Nemours, Inc., Wilmington, DE, USA. It was also found that superior strength and modulus of elasticity are achieved by an isotropic (straight parallel lines) pattern compared with a concentric pattern. Bianchi et al. [7] investigated the effect of process parameters such as extrusion temperature, layer thickness, and printing speed to ensure low energy consumption of printing using short glass fiber-reinforced (GlassPA) and carbon fiber-reinforced (CarbonPA) polyamide composites. The mechanical properties of CarbonPA were found to be superior to those of GlassPA. The more ecologically friendly option for tensile load applications was CarbonPA because of its weight reduction that can be achieved. On the other hand, GlassPA has a lower environmental footprint when the samples are subjected to flexural loads.

Block et al. [8] found that continuous fiber-reinforced nylon components showed a significantly higher tensile strength of 986 MPa and modulus of 64 GPa compared with short fiber-reinforced parts with only 33 MPa and 1.9 GPa, respectively. The authors also suggested printing with a thermoplastic material reinforced with short fibers above the crucial fiber length to address the issues inherent to continuous fiber printing, namely the gaps and precise positioning of fibers. Uşun and Gümrük [9] produced continuous carbon fiber reinforced PLA filament using a melt impregnation line consisting of fiber spreading, polymer mixture, and mold sections. The fused filament fabrication (FFF) printed composites with a fiber content of 40% resulted in a tensile strength of 544 MPa and flexural strength of 310 MPa. This method of composite manufacturing produced good fiber wetting but was limited in the homogeneous distribution of the fibers since the concentration of the fibers was observed in the filament center with the decreasing fiber fraction. A similar study by Wang et al. [10] focused on the development of continuous carbon fiber composites by impregnation of plastics with fibers.

Naranjo-Lozada et al. [11] examined the effect of geometric variables on the tensile performance of chopped carbon fiber reinforced (Onyx^®^, Markforged, Waltham, MA, USA), and CFRP composites. The results concluded that Onyx^®^ samples demonstrated only a slight improvement compared with nylon. Moreover, it was found that a triangular pattern results in higher tensile properties than a rectangular one, while the infill density shows a negligible effect on the tensile performance. As for CFRP specimens, increasing the fiber volume fraction and printing a more comprehensive arrangement for fiber placement enhances the tensile properties. Chacón et al. [12] concluded that flat samples have greater values of tensile and flexural strength and stiffness than on-edge samples and that carbon fiber-reinforced composites have the highest mechanical performance with higher stiffness compared with Kevlar^®^ and glass fibers. The results also indicate that, in most cases, strength and stiffness increase as fiber volume content increases. On the other hand, the level of improvement in mechanical performance decreases as the fiber content increases because of weak bonding between the layers of fiber and nylon and higher levels of defects. Araya-Calvo et al. [13] found that a concentric and equidistant reinforcement configuration and a 0.2444 carbon fiber volume ratio maximized the compressive strength and modulus for carbon-fiber-reinforced polyamide 6 samples. As for the flexural response, the highest flexural properties were obtained at a 0.4893 carbon fiber volume ratio, concentric reinforcement, and XY orientation, which is perpendicular to the applied force. Tian et al. [14] suggest a nozzle temperature of 200 °C–230 °C, layer thickness of 0.4 mm–0.6 mm, and hatch spacing of approximately 0.6 mm to achieve optimal printing of carbon-fiber-reinforced PLA composites. The authors found a maximum flexural strength of 335 MPa and a flexural modulus of 30 GPa with a fiber content of 27%.

Parmiggiani et al. [15] concluded that printing mixed orientations of the continuous carbon fiber can produce balanced laminates that can be exposed to multiaxial stresses. It was also observed that these sequences, particularly the quasi-isotropic layer configurations 0°/+45°/90°/−45 and 0°/90°/+45°/−45°, have a more significant impact on the flexural performance compared with tensile properties. Lupone et al. [16] concluded the highest tensile strength was achieved by the longitudinal fiber arrangement, followed by cross-ply, quasi-isotropic 0°/±60°, and lastly, quasi-isotropic 0°/45°/90°/−45° in continuous carbon fiber reinforced PA composites. El Essawi et al. [17] found that the most significant parameter for the enhancement in the tensile properties of continuous carbon fibers was the fiber deposition angle, followed by infill density, while the position of the carbon fiber layers had a relatively low impact. In the case of the toughness property, on the contrary, the position of the reinforced layers was the most important determinant because of the load-bearing capacity.

Few studies focused on the characterization of fracture toughness of short carbon fiber composites. Papon and Haque [18] observed significant increases of 42% for 0°/90° and 38% for 45°/−45° in the fracture toughness for 5 wt.% CF loading in comparison to neat PLA. The increases in fracture energy were found to be roughly 77% for the 0°/90° and 88% for the 45°/−45° layer orientations. In contrast to the circular nozzle, the square nozzle experiments produced nearly uniform pieces, eliminating the effect of orientation on fracture properties and reducing the inter-bead spaces. Zhang et al. [19] studied the effect of raster pattern and build orientation on fracture toughness and effective fracture energy of Onyx samples, which refer to short carbon fiber-reinforced nylon composites. Authors concluded that while the raster pattern had a negligible effect on toughness, for the build orientation, the XYZ (cross-layer) specimens demonstrated the highest fracture toughness and energy, followed by ZXY (intra-layer) and, subsequently, XZY (inter-layer). In the case of the XZY build orientation, the 0°/90° raster pattern released more fracture energy by 40% than ± 45° samples, whereas an insignificant difference between the two patterns was observed in other orientations. In the study by Kong et al. [20], the carbon fiber layers in the double cantilever beam experiment demonstrated significant resistance to mode I interface failure by fiber bridging, indicating a good printing quality between the two fiber layers. On the contrary, the shear resistance between carbon fiber layers was poor, as shown by the relatively low pure mode II fracture toughness in the end-notched flexure test. Katalagarianakis et al. [21] observed fiber breakage on the mode I and mixed mode I-II surfaces, confirming the occurrence of fiber bridging for mode I loading. A significant amount of energy was dissipated when the crack expanded at the fiber/matrix contact due to intense plastic deformation when loaded in mode II.

Bhandari et al. [22] found that incorporating short carbon fibers in PLA and PETG results in a substantial fall in interlayer tensile strength because of anisotropy. Annealing PLA-CF and PETG-CF composites at 90 °C and 120 °C enhanced the interlayer tensile strength significantly by increasing interlayer bonding and decreasing porosity. Chabaud et al. [23] assessed the hygromechanical properties of carbon and glass-reinforced PA composites. They identified decreases of 18% and 25% of tensile strength and modulus in the longitudinal direction and reductions of 70% and 45% of the corresponding values owing to water sorption by the matrix and moisture-induced delamination. As for the glass composites, 45% and 80% decreases were detected in the tensile strength and modulus of elasticity in the transverse direction, respectively. In the longitudinal direction, although the tensile strength decreased by a quarter compared with the reference sample, the elastic modulus remained approximately consistent across different RH percentages. This is attributable to non-overlapping microstructures in the composite, which mitigates hygroscopic swelling.

Viscusi et al. [24] developed a manufacturing process to obtain hybrid constructions. This process integrates 3D-printed composites using FFF and cold-sprayed metallic layers. All parameters needed to optimize the properties of the parts were identified. It was concluded that this new manufacturing process is promising. Pizzorni et al. [25] investigated the effect of low-pressure plasma treatment on the performance of onyx samples. The shear strength of single-lap joints was almost three times greater than that of abraded samples. The treatment resulted in the generation of more effective interfacial conditions between the adherend and adhesive than the interlaminar ones inside the substrate, indicating the susceptibility of the material to delamination. Saeed et al. [26] studied the effect of varying the amount of carbon fiber in polyamide-based composites and applying pressure to the parts using a platen press for an hour at a constant temperature. Compared with other specimens, the hot-pressed samples with unidirectional fiber orientation had the highest strength and modulus. The samples that were hot-pressed at 130 °C, 50 bar of pressure, and held in the press machine for an hour demonstrated the maximum tensile strength and elastic modulus, indicating an improvement in the adhesion of the layer due to the reduction in porosity. Tang et al. [27] examined 3D-printed continuous CFRP composites with and without designed waved filaments undergoing longitudinal compression tests. When waved filaments are added, there is a noticeable decline in the failure strength from 293 MPa to 181 MPa. However, when additional waved filaments are used, further significant degradation is prevented, resulting in a strength of 160 MPa. Fracture occurs at the cross-section with a maximum misalignment angle of about 7°.

The aim of the work is to study the mechanical properties of continuous carbon fiber-reinforced PETG composites with different reinforcement angles and number of layers. Due to the specimens’ width size and the printer’s limitations, only 0°, +15°, and −15° printing angles of the CCF were considered. In this work, the tensile and flexural performance of specimens was investigated. This study differs from the available literature because a minimal amount of carbon fiber reinforcement was used for cost reduction. Furthermore, cost and printing time analyses were conducted in addition to mechanical characterization.

## 2. Materials and Methods

This section provides an overview of the materials used for the experiment, the printing parameters, and the different testing methods used to investigate the mechanical properties of the specimens.

### 2.1. Materials and Manufacturing Parameters

Clear PETG and continuous fiber co-extrusion (CFC) PETG with continuous carbon fiber (CCF) by Anisoprint were the materials utilized in the experiment. The polyether-based matrix, Clear PETG, provides excellent durability, low shrinkage, and interlayer bonding to produce thin-walled prints [28]. The high-strength reinforcement CCF 1.5k + CFC PETG is printed through the co-extrusion of carbon fiber with a diameter of 0.35 mm and CFC PETG with a plastic filament diameter of 1.75 mm [29]. The FFF technology used for manufacturing plastic and composite specimens is Anisoprint Composer A4, a desktop CFC 3D printer with a closed chamber. The printer is equipped with a dual nozzle print head, particularly the FFF extruder and CFC extruder, which have reinforced filament-cutting devices. The diameter of nozzles is 0.4 mm [30].

SolidWorks 2022, a computer-aided design (CAD) software, was used to design the testing specimens. The CAD file was then converted to the stereolithography (STL) format to specify the surface geometry of the model. The model was sliced in Aura v2.4.8 and transformed into a G-Code file. The printing parameters selected for the specimens are shown in Table 1.

### 2.2. Tensile Test—DOE

**Figure 1:**
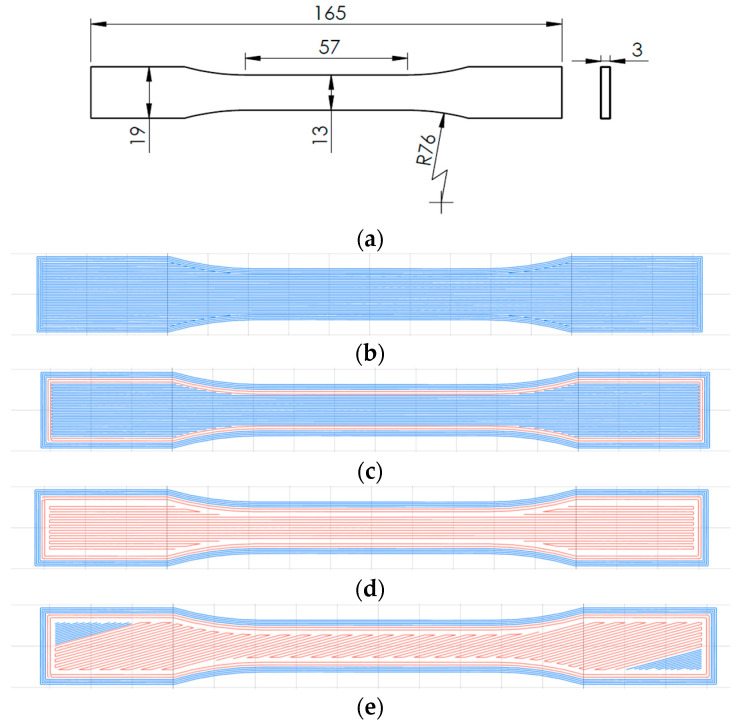

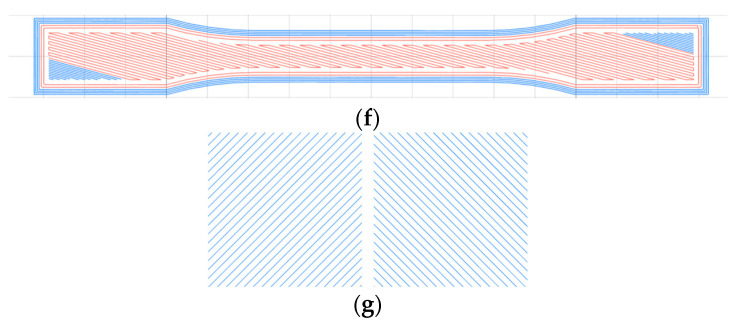
(**a**) Tensile test specimen; (**b**) PETG specimen; (**c**) Contour only specimen; (**d**) Infill guide 0°; (**e**) Infill guide 15°; (**f**) Infill guide −15°; (**g**) Plastic infill pattern.

Figure 1a shows that the specimens were manufactured following the ASTM D638 standard [31] for tensile testing. The PETG specimen was printed as a reference for comparison with the carbon fiber-reinforced specimens. The external shell and plastic parameters (shown in blue) and the longitudinal infill pattern (in blue) used for the plastic are shown in Figure 1b. The “contour only” specimen with two carbon fiber-reinforced perimeters (in red) and the plastic with a longitudinal infill pattern (in blue) are shown in Figure 1c. The 0° specimen with two carbon fiber-reinforced perimeters and carbon fiber-reinforced infill with a 0° guide angle (in red) is shown in Figure 1d. The reinforced infill pattern is solid. The 15°/−15° specimen layers with two carbon fiber-reinforced perimeters and carbon fiber-reinforced infills with 15° and −15° guide angles (in red) are shown in Figure 1e,f, respectively. The edges (in blue) inside the CCF perimeters were unreinforced PETG due to the minimum fiber line length (20 mm) that Anisoprint can print. The plastic infill pattern of matrix PETG was zigzag, with 100% infill density, as shown in Figure 1g.

Due to the size of the ASTM specimen and the limitation of the printer, only angles up to ±15° were considered. Indeed, to lay down a continuous line of carbon fiber, its minimum length should be at least 20 mm; otherwise, the printer cannot deposit the fiber. For the ASTM specimen considered, if the printing angle is above 18°, this criterion is not fulfilled anymore, and the printer cannot print the fiber.

**Figure 2:**
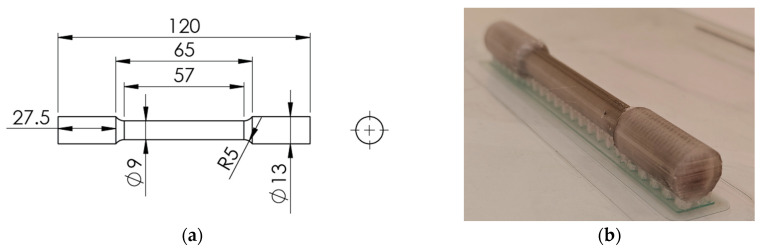
Cylindrical tensile test specimen (**a**) and printed specimen with its built supports (**b**).

Table 2 summarizes the types of tensile test specimens examined in the. The cylindrical tensile test specimens were printed with full carbon fiber reinforcement in the longitudinal direction. The dimensions of the specimens and a printed specimen with its built support are shown in Figure 2.

Three replicas for each type of sample were printed and tested. The specimens were subjected to the tensile test using the Liagong CM-50 universal testing machine, which has a maximum load capacity of 50 kN. The crosshead rate of 1 mm/min was applied to all specimens. An electromechanical extensometer with a 25 mm gauge length was used to measure the elongation.

### 2.3. Flexural Test—DOE

**Figure 3:**
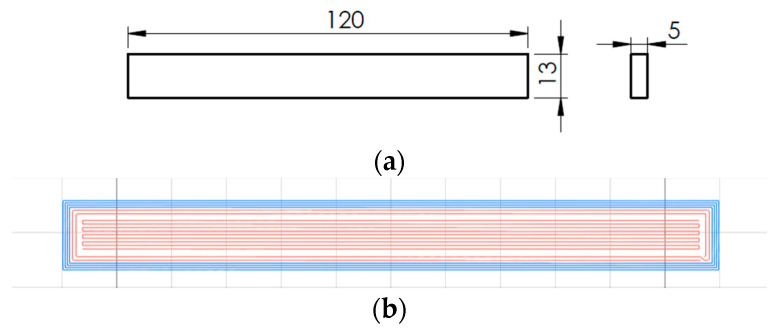
(**a**) Flexural test specimen; (**b**) Infill guide 0°.

Figure 3a shows that the specimens were manufactured following the ASTM D790 standard [32] for flexural testing.

Figure 3b shows the 0° specimen with two carbon fiber-reinforced perimeters and carbon fiber-reinforced infill with a 0° guide angle (in red). The reinforced infill pattern is solid.

Four types of specimens were investigated: (1) PETG without reinforcement, (2) one layer of carbon fiber-reinforced below the neutral axis, (3) two layers of carbon fiber-reinforced below the neutral axis, and (4) four layer of carbon of reinforcement distributed evenly along the whole thickness. For the specimens with one layer, the CCF was added just above the floor (this is defined by the minimum number of layers of PETG that must be printed before one layer of CCF can be printed, in this case, 0.84 mm); this ensures a maximum increase in the stiffness of the cross-section. Based on the Aura preprocessor, another layer of CCF can only be added 0.32 mm above the previous layer. This is due to the “microlayer” height parameter that is set to 0.32 mm and the fact that the initial diameter of the CCF is 0.35 mm. It was decided to have one layer of 0.32 mm of PETG between the first and second layers of CCF to ensure that the composite effect can develop fully. Therefore, the second layer was located at 1.16 mm above the platform. The two top reinforcement layers were located approximately symmetrically from the neutral axis. The locations of these layers were approximately symmetrically as there is a limitation of the macrolayer of 0.32 mm.

The types of flexural test specimens examined in this study are summarized in Table 3. The position(s) of the CCF layer (s) within the cross-section is also provided. Three replicas for each type of sample were printed and tested. The specimens were subjected to the flexural test using the MTS Criterion 40 universal testing machine with a maximum load capacity of 1 kN. The crosshead rate of 1 mm/min was applied for all specimens. The span length of 80 mm was used to have the support span-to-depth ratio of 16:1.

### 2.4. Scanning Electron Microscopy

The morphological characteristics of the specimens were examined using the scanning electron microscope (SEM) Jeol JCM-7000 Benchtop SEM, JEOL Ltd., Akishima, Tokyo, Japan. An Au coating was applied to the specimens to be observed in high vacuum mode.

**Figure 4:**
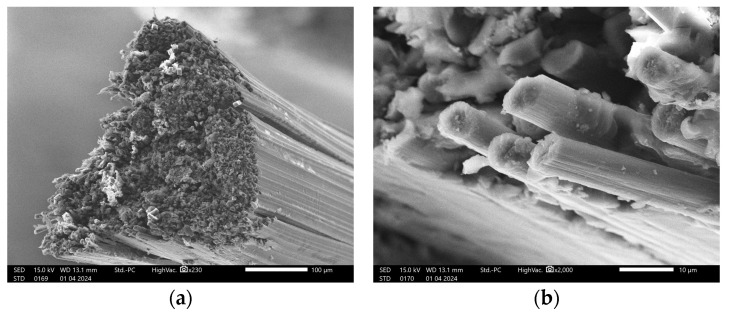
SEM of the carbon fiber (**a**) bundle; (**b**) individual fibers.

Figure 4 shows the SEM analysis of the single strand of carbon fiber before printing. The cracks along the longitudinal direction were due to the cutting of the fiber with the scissors. The resin effectively impregnated the individual fibers within the strand. The fiber bundle has a high-volume fraction ratio of highly dense fiber with low porosity.

## 3. Results and Discussion

This section reports the findings from the experiments, particularly tensile and flexural tests.

### 3.1. Tensile Test Results

**Figure 5:**
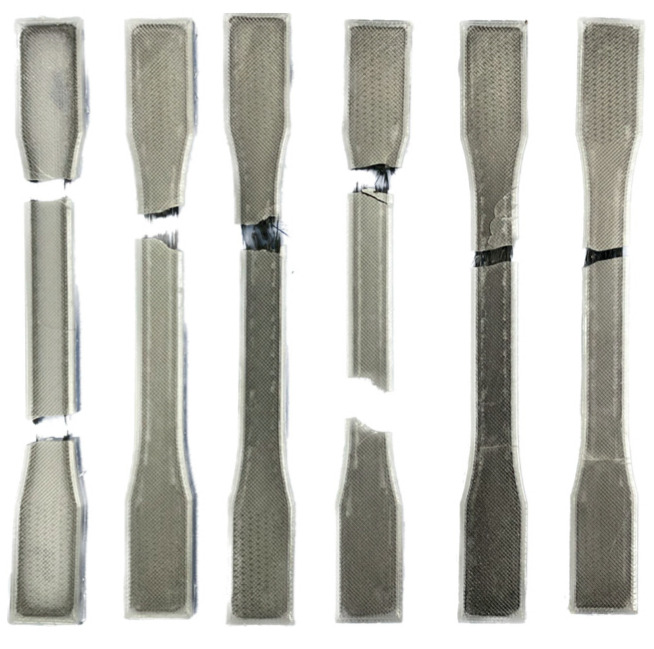
Fracture sites of the flat tensile test specimens.

Based on the rupture sites of the specimens shown in Figure 5, it can be observed that PETG exhibits ductile behavior, whereas the addition of the carbon fiber reinforcement leads to the transition to a brittle fracture. The contour only specimen saw the matrix and fiber contours breakage at both sites near the increase in the cross-sectional area. The 0° sample experienced the failure of fibers and matrix along the gauge length. A similar fracture was observed in the 15°/−15° and 0°/0° specimens. For the samples with three layers of carbon fiber reinforcement, the failure occurred because of the fracture of the matrix, as the fibers are seen holding the two parts of the specimen together.

**Figure 6:**
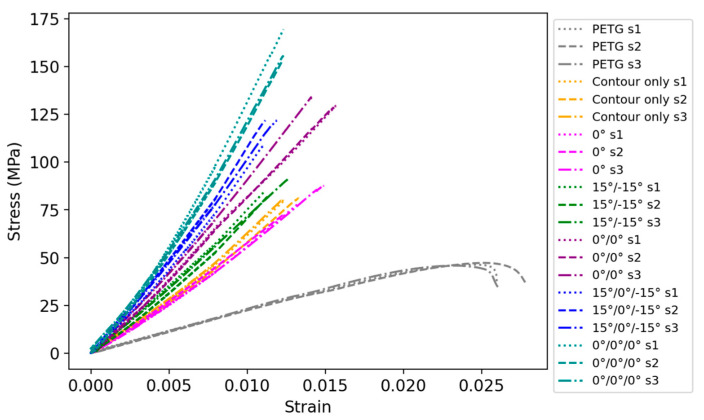
Stress–strain curve of the tensile test specimens.

Figure 6 shows the experimental stress–strain curves of the PETG and composite specimens with different numbers of carbon-reinforced layers and guide directions. The addition of carbon reinforcement significantly improves the tensile strength while decreasing ductility, resulting in the brittle failure of all composite specimens.

**Figure 7:**
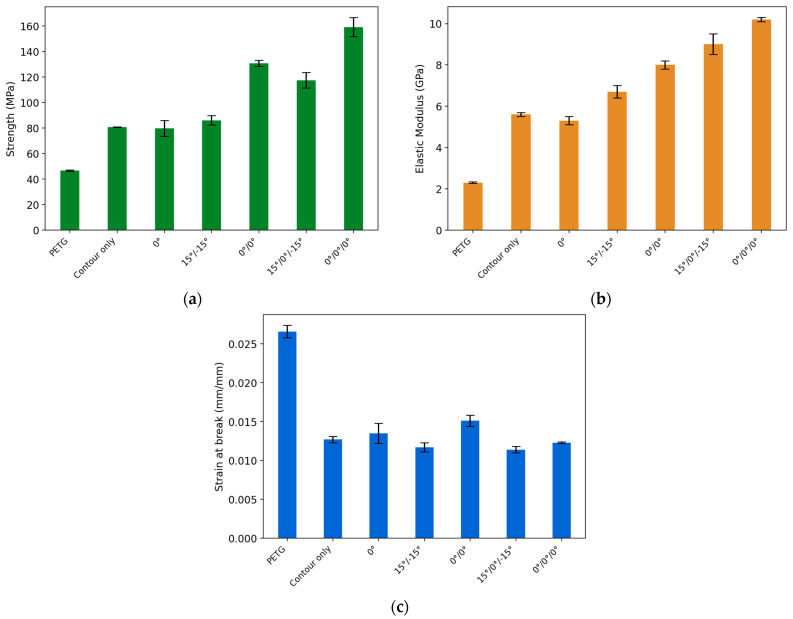
Tensile test results: (**a**) Tensile strength; (**b**) Elastic modulus and (**c**) strain at break.

Using experimental data in Figure 6, the values of tensile strength, elastic modulus, and strain at break were calculated and are shown in Table 4 and Figure 7. Table 4 summarizes the tensile test results with the average (Mean) and standard deviation (SD) values. Figure 7a compares the ultimate tensile strength of the PETG and carbon-fiber-reinforced composite samples. The failure of the PETG reference specimen exhibited a tensile strength of 46.5 MPa. Adding one layer of the PETG/CCF layer with a 0° guide angle resulted in a strength value of 79.8 MPa, a 71.6% increase from the reference. The contour-only specimen provided an almost equivalent strength of 80.7 MPa (73.5%). As for the samples with two layers of reinforcement, 15°/−15° showed a value of 86.0 MPa (84.9%), while a 180.9% rise was observed for the 0°/0° composite sample displayed 130.6 MPa. The 15°/0°/−15° provided the tensile strength value of 117.4 MPa, which is 152.5% higher than the reference. Among the test specimens, the highest tensile strength of 159.1 MPa (242.2%) was demonstrated by the composite with three layers of reinforcement and a 0° guide angle. Overall, it can be observed that the specimens with a 0° guide angle exhibited significantly higher ultimate tensile strength in comparison to the 15° ones for the specimens with the two and three layers of CCF reinforcement. A more moderate increase from the PETG was reported for the composite samples with 15°/−15° and 15°/0°/−15° guide angles. The contour-only specimen provided the same tensile strength as the one-layer 0° specimen.

Figure 7b shows the modulus of elasticity of the specimens with the values showing a similar trend to the ultimate strength. For the reference sample, the elastic modulus of 2.3 GPa was detected, whereas a significant increase in stiffness was observed for all composites. The contour-only specimen provides 5.6 GPa (143.5%), while a similar value of 5.3 GPa, a 130.4% increase, was noted for the 0° specimen. The specimens with two layers of carbon fiber reinforcement demonstrated a tensile modulus of 6.7 GPa for 15°/−15° (191.3%) and 8.2 GPa for 0°/0° (256.5%). The largest stiffness was recorded for the cases with three layers of reinforcement. The specimen with a 15°/0°/−15° guide angle saw a 291.3% increase in the modulus of elasticity equivalent to 9.0 GPa, while the 0°/0°/0° exhibited a rise to 10.2 GPa (343.5%).

Regarding the strain at break (Figure 7c), the reference elongated for PETG was 0.0266, the largest deformation among the tested specimens because of the ductility of the PETG material. The reinforcement of contours with CCF resulted in a brittle failure mode with a strain of 0.0127 (−52.3%). The 0° specimen demonstrated the elongation to 0.0135 (−49.2%). The 0° guide angle allowed slightly more elongation compared with the 15°, while a higher percentage of the carbon fiber reinforcement reduced the strain at break. The 15°/−15° specimen showed the 0.0117 strain at break (−56.0%), whereas the 0°/0° broke at the strain of 0.0151 (−43.2%). The 15°/0°/−15° sample had the lowest strain at break equivalent to 0.0114 (−57.1%), and the 0°/0°/0° specimen resulted in a value of 0.0123 (−53.8%). The reason for the more brittle behavior of 15°/−15° and 15°/0°/−15° specimens is that for a given overall sample elongation, fibers oriented at a 15-degree angle to the loading direction are subjected to a higher elongation. The composite fails at a lower strain (or elongation) due to the higher elongation of the fibers at a 15-degree angle. Overall, from Figure 6 and Figure 7 and Table 4, it can be concluded that the experimental results are in good agreement with other publications. For example, the increase in strength and stiffness and decrease in strain at break, as a function of the amount of CCF, agrees with the findings from [12,15]. The variation in terms of print angles of the CCF is in good agreement with the findings from [16].

**Figure 8:**
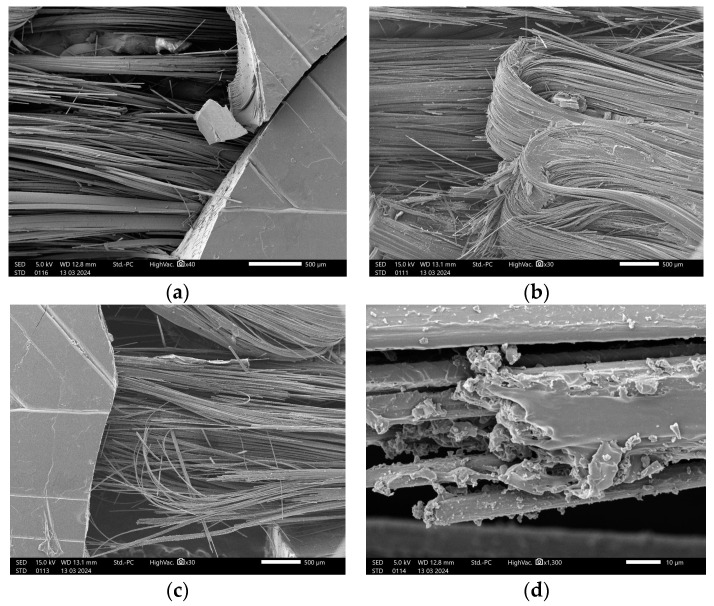
SEM of the rupture site of the 15°/0°/−15° specimen (**a**) fiber rupture; (**b**) curling of fibers; (**c**) fiber pullout; (**d**) fiber strands.

The SEM of the rupture site of specimens was conducted to investigate the failure modes. In the case of 15°/0°/−15°, the samples experienced the transverse rupture of the matrix PETG. The failure occurred because of the breakage of the fibers and matrix (see Figure 8a). The bridging of the fibers was observed as the two parts of the specimen were still attached. Some fibers were curled, as shown in Figure 8b, due to the printing process fiber path of 15° and −15° guide angles and the fracture of these layers was also recorded. As depicted in Figure 8c, elongation and fiber pullout also occurred. Plastic residue on the fibers and fibers’ pullout were observed in Figure 8d.

**Figure 9:**
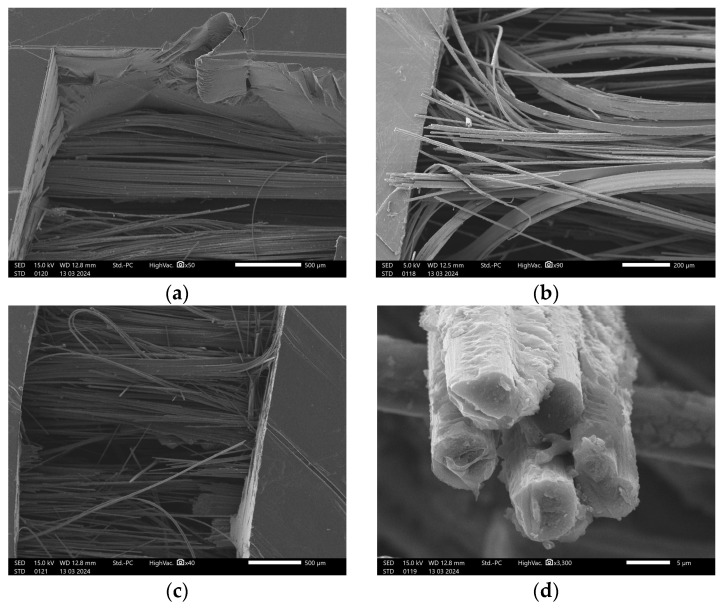
SEM of the rupture site of the 0°/0°/0° specimen (**a**) matrix fracture and debonding; (**b**) fiber pullout and rupture; (**c**) fiber bridging; (**d**) fiber strands.

Similarly, the SEM examination of the 0°/0°/0° specimen resulted in the breakage of the PETG plastic matrix and debonding of the matrix and fibers, as seen in Figure 9a. The fiber pullout and fiber rupture are shown in Figure 9b. As shown in Figure 9c, the bridging of the fibers between two parts of the matrix was present. Figure 9d shows the image of the fiber strands.

### 3.2. Cylindrical Tensile Test

**Figure 10:**
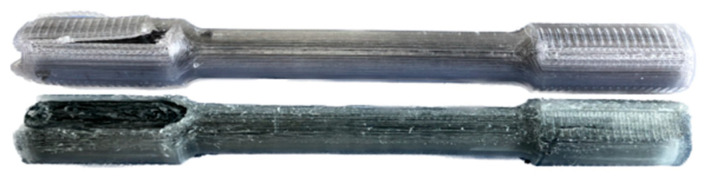
Delamination of the tensile test specimens.

The first batch of specimens shown in Figure 10 showed delamination in the gripping section of the matrix. The test was stopped because of samples slipping from the tensile test jaws and the deformation of the gripping section. Consequently, the gripping section’s design was modified to increase the surface between this CCF layer and the PETG outer layer.

**Figure 11:**
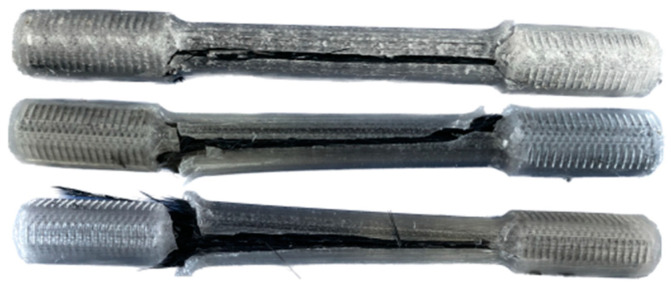
Fracture sites of the cylindrical tensile test specimens.

The results of the fracture of the cylindrical test samples are given in Table 5. As shown in Figure 11, the matrix fracture occurred along the sample’s longitudinal axis. During the experiment, cracking sounds were heard, corresponding to the transverse propagation of cracks within the cross-section. This is followed by a louder sound with a drop in load-carrying capacity and the failure of the specimen due to delamination of the CCF. The debonding of the matrix and fiber interface is shown in Figure 11. The specimens failed at an average ultimate tensile strength of 155.0 MPa and strain of 0.0073. The stiffness of tensile specimens was recorded to be 15.1 GPa.

**Figure 12:**
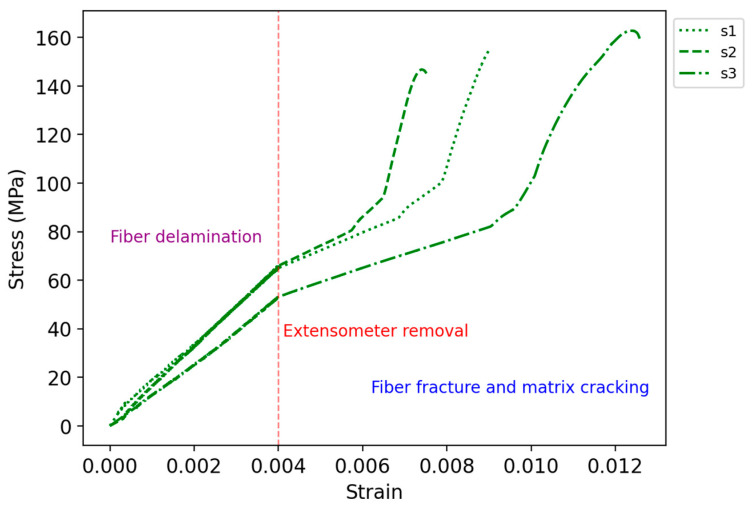
Stress–strain curve of the cylindrical tensile test specimens.

Figure 12 shows the stress–strain curves. The specimens first experienced interlayer shear stress, which resulted in fiber delamination. After removing the extensometer at the strain of 0.004, a change in the slope was observed. This is due to the change in the measurement/calculation of the strain in the tensile test machine and can be considered as an artifact. The failure occurred because of fiber fracture and matrix cracking.

### 3.3. Flexural Test Results

The results from the three-point bending test are summarized in Table 6. In the case of the reference PETG specimen, the maximum load was equivalent to 215.4 N. The embedding of one carbon fiber-reinforced layer below the neutral axis increased the flexural load to 285.1 N, a 32.4% increase from the reference. The specimen with two layers below the neutral axis provided an almost equivalent load of 288.1 N (33.8%), as for the samples with four layers of reinforcement distributed evenly along the whole thickness, a value of 278.9 N (29.5%) was recorded for the maximum load. Overall, it can be observed that increasing the number of CCF layers had no significant effect on the maximum load of composites.

**Figure 13:**
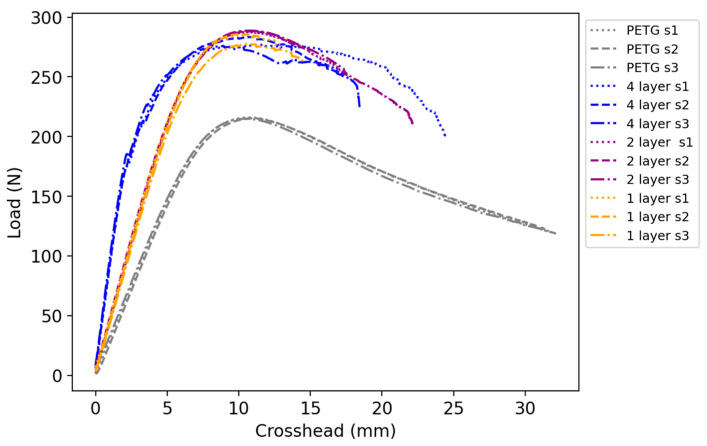
Load-displacement curve of the flexural test specimens.

As for the stiffness, the PETG samples showed a value of 29.1 N/mm. The one-layer specimen had a stiffness of 41.7 N/mm, an increase of 43.3% from the plastic sample, while a similar value of 43.1 N/mm (48.1%) was recorded for the two-layer specimen. On the contrary, the specimen with four layers of CCF showed a 198.6% increase from the reference with a stiffness of 86.9 N/mm. The load-displacement curves of the flexural test specimens are shown in Figure 13.

It can be seen that the PETG specimen had the lowest stiffness, as expected. The samples with one and two layers of reinforcement have similar stiffness and maximum load values. This is likely because the section is subjected to compression plasticization; the neutral axis shifts closer to the layer of CCF, which is slightly below the neutral axis, reducing its contribution to the stiffness of the samples. An increase in stiffness was observed for the samples with four layers, but the maximum load was slightly decreased. The addition of the CCF can explain the layers’ increase in stiffness. The outer layers, close to the top and bottom surface, have the highest contributions concerning the stiffness of the specimens with 1-, 2-, and 4-layers. The inner layers of the specimens with 2- and 4-layers have limited effect on the stiffness of the specimens as they are close to the neutral axis and, therefore, are not subjected to high strain and stress. This explains why the 1- and 2-layer samples have similar stiffness values. Once the specimens are subjected to large deflections, the CCF reaches their ultimate strain and starts to fail. Therefore, the inner CCF takes more strain and stress. This allows the specimens to experience larger deformations (crosshead displacement) before the specimen fails. It can be seen in Figure 13 that the specimens with 2- and 4-layers have higher crosshead displacement than the 1-layer samples. In conclusion, the increase in stiffness of specimens in bending, which can be seen in Figure 13 and Table 6, because of the increased amount of CCF reinforcement, is in line with the results from [12,13,15].

### 3.4. Cost and Time Analysis

A manufacturing time and cost analysis was performed using data obtained from the Aura Anisoprint slicer, Aura. The approximate costs of one spool are Clear PETG-60.0 €, CFC PETG-60.0 €, and CCF-300.0 €. Table 7 and Table 8 show the estimated cost of the raw material needed to print the part (PETG + CCF); the printing time has also been calculated, as well as the amount of CCF needed in meters. From a cost viewpoint, as the amount of CCF is increased, the cost of the part also increases, this is due to the fact the CCF is significantly more expensive than PETG. As can be seen from Table 7, tensile specimens with PETG are over four times cheaper than the ones with three layers or reinforcement. Similarly, as shown in Table 8, PETG specimens are three times cheaper for flexural specimens than the ones with three layers of CCF.

Additionally, as CCF is added to the part, the printing time and cost increase dramatically. For tensile specimens, the time almost doubles from just above 1 h to almost 2 h. For flexural specimens, the printing time increases by 55% (from 1 h 3 min to 1 h 38 min). This increase in manufacturing time is due to the fact that when CCF is used, the printer takes time to heat each extruder when the printed material is changed, and the printing speed of CCF is lower than PETG.

In the case of the tensile test specimens, similar values of tensile strength were recorded for the contour only (80.7 MPa) and 0° (79.8 MPa), and the same printing time of 1 h 35 min was taken for the cost of 1.8 € and 2.0 €, respectively. The “contour only” specimen used 2.3 m of CCF, while the 0° specimen had 2.9 m of CCF. The elastic modulus of the contour only (5.6 GPa) and 0° (5.3 GPa) were also similar.

For the specimens with two layers of reinforcement, 15°/−15° and 0°/0° have almost the same amount of CCF equivalent to 5.5 m and 5.6 m, respectively, with a cost of 3.0 €. However, 0°/0° provides considerably higher tensile strength (130.6 MPa) and modulus (8.0 GPa) than 15°/−15° (86.0 MPa and 6.7 GPa). The 0°/0° specimen takes slightly less printing time at 1 h 49 min compared with 15°/−15° at 1 h 46 min.

A similar trend was observed for the specimens with three layers of CCF reinforcement. For the almost identical cost of 4.0 € and 4.1 €, respectively, 15°/0°/−15° had a tensile strength of 117.4 MPa and elastic modulus of 9.0 GPa, while 0°/0°/0° showed a strength of 159.1 MPa and modulus of 10.2 GPa. The printing times of 15°/0°/−15° and 0°/0°/0° are 1 h 57 min and 1 h 54 min, respectively.

For the flexural test samples, it was also found that the strength of a part may not be increased by increasing the amount of CCF, but its stiffness will be increased. The 1-layer specimen showed a maximum load of 285.1 N and a stiffness of 41.7 N/mm with a printing time that took 1 h 15 min. The amount of CCF is 1.6 m, and the cost is 1.5 € for these specimens. The 2-layer specimen had an increase in the printing time to 1 h and 23 min as well as an almost doubling in the amount of CCF to 3 m, while the load reached only 288.1 N and stiffness was equivalent to 43.1 N/mm. The cost of printing is 2.0 €. The 4-layer specimen used 5.8 m of CCF and cost 3.1 €. The maximum load was almost equivalent, 278.9 N, whereas the stiffness increased twice to 86.9 N/mm. The printing time increased to 1 h and 38 min.

As it was shown in Section 3.1 and Section 3.3, the material properties do not increase proportionally to the amount of CCF used in the parts. Therefore, it is suggested that the end users consider:

1. The strength of the parts needed based on their loading;
2. The cost of the raw materials and printing time to calculate the full cost of the part;
3. Consider using the “contour” option when printing to strengthen the inner and outer parts of the specimen as well as any holes that may be needed, as this is the best way to strengthen these areas.

## 4. Conclusions

This work investigated the mechanical performance of PETG and CCF-reinforced composites with different guide angles and several continuous carbon-reinforced layers. The tensile properties of the composites with different reinforced infill guide angles and number of layers were examined. The flexural specimens with different CCF layer counts and positions were tested. Moreover, an analysis of manufacturing time and cost was conducted. The following conclusions can be made:

- In the case of tensile test specimens, adding one layer of the CCF reinforcement with a 0° guide angle increased the strength by more than double the reference PETG, while the contour-only specimen provided an almost equivalent strength.
- For the specimens with two and three layers of CCF reinforcement, the specimens with a 0° guide angle showed a significantly greater ultimate tensile strength than the specimens with a 15° one. In the case of the composite samples with guide angles of 15°/−15° and 15°/0°/−15°, a more moderate rise from the PETG was observed.
- 0°/0° offers higher tensile strength and elastic modulus in comparison to 15°/−15°, despite an equivalent cost and amount of CCF used. The same phenomenon was observed for 15°/0°/−15° and 0°/0°/0°.
- In the case of flexural test specimens, adding more CCF increases the stiffness of a part but not necessarily its strength.
- The PETG specimen, as predicted, has the lowest stiffness, as can be observed. The stiffness and maximum load of the samples with one and two reinforcing layers are comparable. There was a noticeable increase in stiffness but a slight drop in the maximum load for the samples with four layers.
- The use of CCF increases the printing time due to the lower printing speed for CCF than PETG and the fact that the extruder needs to be heated when the material is changed.
- As CCF is significantly more expensive than PETG, the end consumer may consider a trade-off between cost, printing time, and strength of the parts while designing a part.

This study concentrated on the tensile and flexural performance of printed composites with the consideration of economic parameters. Future work can focus on studying the fatigue resistance, the bonding strength between the outer layer of polymers and the first layer of CCF, the impact resistance of the composites, and the fracture toughness; there are essential properties that need to be investigated to improve the reliability, durability, and impact resistance of FFF-printed continuous carbon fiber reinforced composites. Limited publications are available on these topics in the literature.

## Figures and Tables

**Table 1:** Printing parameters.

Parameters	Value
Macrolayer height (mm)	0.32
External shell layer height (mm)	0.08
Infill layer height (mm)	0.16
Plastic perimeters layer height (mm)	0.16
Thick support layer height (mm)	0.16
Extrusion width (mm)	0.65
Infill density (%)	100
Plastic extrusion (°C)	240
Composite extrusion (°C)	235

**Table 2:** Types of tensile test specimens.

Specimen	Number of Carbon Fiber Layers
PETG	None
Contour only	3
0°	1
15°/−15°	2
0°/0°	2
15°/0°/−15°	3
0°/0°/0°	3

**Table 3:** Types of flexural test specimens.

Specimen	Number of CCF Layers	Distance from the Bottom Surface (mm)
PETG	No CCF	NA
1 layer	One CCF layer below the neutral axis	0.84
2 layers	Two CCF layers below the neutral axis	1st layer: 0.84 2nd layer: 1.16
4 layers	Four CCF layers distributed evenly along the whole thickness	1st layer: 0.84 2nd layer: 1.16 3rd layer: 3.4 4th layer: 3.72

**Table 4:** Tensile test results.

	Tensile Strength (MPa)		Elastic Modulus (GPa)		Strain at Break (mm/mm)	
	Mean	SD	Mean	SD	Mean	SD
PETG	46.5	0.6	2.3	0.03	0.0266	0.0008
Contour only	80.7	0.2	5.6	0.1	0.0127	0.0004
0°	79.8	6.2	5.3	0.2	0.0135	0.0013
15°/−15°	86.0	3.7	6.7	0.3	0.0117	0.0006
0°/0°	130.6	2.4	8.0	0.2	0.0151	0.0007
15°/0°/−15°	117.4	6.1	9.0	0.5	0.0114	0.0004
0°/0°/0°	159.1	7.5	10.2	0.1	0.0123	0.0001

**Table 5:** Tensile test results of cylindrical specimens.

	Tensile Strength (MPa)		Elastic Modulus (GPa)		Strain at Break (mm/mm)	
	Mean	SD	Mean	SD	Mean	SD
Cylinder	155.0	6.5	15.1	1.5	0.0073	0.0018

**Table 6:** Flexural test results.

	Load (N)		Stiffness (N/mm)	
	Mean	SD	Mean	SD
PETG	215.4	0.5	29.1	0.1
1 layer	285.1	0.5	41.7	1.2
2 layers	288.1	0.5	43.1	0.8
4 layers	278.9	3.4	86.9	1.3

**Table 7:** Tensile test specimens.

Specimen	Cost (EUR €)	Time	Amount of CCF (m)
PETG	0.9	1 h 7 min	None
Contour only	1.8	1 h 35 min	2.3
0°	2.0	1 h 35 min	2.9
15°/−15°	3.0	1 h 49 min	5.5
0°/0°	3.0	1 h 46 min	5.6
15°/0°/−15°	4.0	1 h 57 min	8.2
0°/0°/0°	4.1	1 h 54 min	8.3

**Table 8:** Flexural test specimens.

Specimen	Cost (EUR €)	Time	Amount of CCF (m)
PETG	0.9	1 h 3 min	None
1 layer	1.5	1 h 15 min	1.6
2 layers	2	1 h 23 min	3
4 layers	3.1	1 h 38 min	5.8

## Data Availability

The raw data supporting the conclusions of this article will be made available by the authors on request.
